# A Hierarchical View Pooling Network for Multichannel Surface Electromyography-Based Gesture Recognition

**DOI:** 10.1155/2021/6591035

**Published:** 2021-08-26

**Authors:** Wentao Wei, Hong Hong, Xiaoli Wu

**Affiliations:** ^1^School of Design Arts and Media, Nanjing University of Science and Technology, Nanjing, Jiangsu, China; ^2^School of Electronic and Optical Engineering, Nanjing University of Science and Technology, Nanjing, Jiangsu, China

## Abstract

Hand gesture recognition based on surface electromyography (sEMG) plays an important role in the field of biomedical and rehabilitation engineering. Recently, there is a remarkable progress in gesture recognition using high-density surface electromyography (HD-sEMG) recorded by sensor arrays. On the other hand, robust gesture recognition using multichannel sEMG recorded by sparsely placed sensors remains a major challenge. In the context of multiview deep learning, this paper presents a hierarchical view pooling network (HVPN) framework, which improves multichannel sEMG-based gesture recognition by learning not only view-specific deep features but also view-shared deep features from hierarchically pooled multiview feature spaces. Extensive intrasubject and intersubject evaluations were conducted on the large-scale noninvasive adaptive prosthetics (NinaPro) database to comprehensively evaluate our proposed HVPN framework. Results showed that when using 200 ms sliding windows to segment data, the proposed HVPN framework could achieve the intrasubject gesture recognition accuracy of 88.4%, 85.8%, 68.2%, 72.9%, and 90.3% and the intersubject gesture recognition accuracy of 84.9%, 82.0%, 65.6%, 70.2%, and 88.9% on the first five subdatabases of NinaPro, respectively, which outperformed the state-of-the-art methods.

## 1. Introduction

As a noninvasive approach of establishing links between muscles and devices, the surface electromyography- (sEMG-) based neural interface, also known as the muscle computer interface (MCI), has been widely studied in the past decade. Surface electromyography is a type of biomedical signal recorded by noninvasive electrodes placed on human skin [1]; it is the spatiotemporal superposition of motor unit action potential (MUAP) generated by all active motor units (MU) at different depths within the recording area [2]. sEMG recorded from subject's forearm measures muscular activity of his/her hand movements, thus, can be used for hand gesture recognition. So far, the sEMG-based gesture recognition techniques have been widely applied in rehabilitation engineering [3–5] and human-computer interaction [6–8].

From the perspective of signal recording, there are two types of sEMG signals: (1) high-density sEMG (HD-sEMG) [9–11] signals which are recorded by electrode arrays that consist of dozens, or even hundreds of electrodes arranged in a grid; (2) multichannel sEMG signals [12, 13] which are recorded by several sparsely located electrodes. For MCIs such as robotic hand prostheses and upper-limb rehabilitation robots, one of the key challenges is to precisely recognize the user's gestures through sEMG signals collected from his/her forearm. Over the past five years, feature learning approaches based on convolutional neural networks (CNNs) have shown promising success in HD-sEMG-based gesture recognition, that is, achieving >90% recognition accuracy in classifying a large set of gestures [11], and almost 100% recognition accuracy in classifying a small set of gestures [14, 15], because HD-sEMG signals contain both spatial and temporal information of muscle activity [16]. Compared to conventional feature engineering approaches based on shallow learning models, a major advantage of feature learning approaches is that the end-to-end learning capability of deep learning models enables them to automatically learn representative deep features from raw sEMG signals without any hand-crafted feature [17].

On the other hand, achieving high accuracy in multichannel sEMG-based gesture recognition performance remains a challenging task, because multichannel sEMG is noisy, random, nonstationary [18], and vulnerable to electrode shift [16] and contains much less spatial information about muscle activities than HD-sEMG [19]. So far, researchers have tried a variety of strategies to improve the multichannel sEMG-based gesture recognition performance, including extracting more representative features [20], using multimodal gesture data collected from multiple sensors [21], and developing more sophisticated deep learning models [15].

In recent years, there has emerged a trend in combining deep learning models with feature engineering techniques, as well-designed time domain (TD) [22], frequency domain (FD) [23], and time-frequency domain (TFD) [24] features have achieved remarkable success in multichannel sEMG-based gesture recognition systems. For example, Zhai et al. [25] calculated spectrograms of sEMG and used them as features for CNN-based gesture recognition and achieved 78.7% gesture recognition accuracy for recognizing 49 gestures. Hu et al. [26] extracted the Phinyomark feature set [23] from raw sEMG signals and fed them into an attention-based hybrid convolutional neural network and recurrent neural network (CNN-RNN) architecture for gesture recognition; they achieved 87% recognition accuracy for recognizing 52 gestures. Betthauser et al. [27] proposed the encoder-decoder temporal convolutional networks (ED-TCN) for sEMG-based sequential movement prediction; the inputs of their proposed ED-TCN model were composed of mean absolute value (MAV) sequences. Chen et al. [28] used continuous wavelet transform (CWT) to process the data as the input of their proposed CNN model.

In machine learning, multiview learning refers to learning from data described by different view-points or different feature sets [29, 30]. On this basis, Wei et al. [31] proposed a multiview CNN (MV-CNN) framework that constructs images generated from different sEMG features into multiview representations of multichannel sEMG. Compared to prior works that combined deep learning models with feature engineering techniques, one of the key characteristics of MV-CNN is that it adopts a “divide-and-aggregation” strategy that is able to independently learn deep features from each individual view of multichannel sEMG. The MV-CNN framework showed promising success in multichannel sEMG-based gesture recognition, as the gesture recognition accuracy achieved by MV-CNN significantly outperformed the state-of-the-art deep learning approaches.

From the perspective of multiview learning, there are generally two types of features, namely, the “view-specific feature” or “private feature” particular for each individual view and the “view-shared feature” or “public feature” shared by all views [32]. The independent learning under each individual view is able to learn view-specific features [33]; on the other hand, it is unable to learn shared information across different views [34]. The MV-CNN framework [31] did consider view-shared learning by an early fusion strategy that concatenates the output from the lowest convolutional layers of all view-specific CNN branches. However, from our perspective, the early fusion strategy used in MV-CNN is still a naive approach based on concatenation; it also ignores the original input feature spaces of different views.

Aiming at improving multichannel sEMG-based gesture recognition via better learning of view-shared deep features, in this paper, we proposed a hierarchical view pooling network (HVPN) framework, in which view-shared feature spaces were hierarchically pooled from multiview low-level features for view-shared learning. In order to build up more discriminative view-shared feature spaces, we proposed a CNN-based view pooling technique named the feature-level view pooling (FLVP) layer, which is able to learn a unified view-shared feature space from multiview low-level features. Compared to MV-CNN [31], the application of hierarchical view pooling and FLVP layer results in a wider (i.e., with more CNN branches) and deeper (i.e., with more convolutional layers in the view-shared learning branches) network architecture, respectively, thus enabling the learning of more representative view-shared deep features.

The remainder of this paper is organized as follows.  Section 2 formulates the multiview learning problem, describes the databases, and details the proposed HVPN framework.  Section 3 introduces the experiments in this paper and provides the experimental setup.  Section 4 presents and discusses the experimental results. Finally, Section 5 concludes the paper.

## 2. Materials and Methods

### 2.1. Problem Statement

According to Wei et al. [31], the problem of multiview deep learning-based gesture recognition using multichannel sEMG signals can be formulated as

(1) $$\begin{array}{c} y=H\left(v_{1},v_{2},\ldots ,v_{n};\theta \right), \end{array}$$

where *v*_1_, *v*_2_,…, *v*_*n*_ denote multiview representations from *n* different views of *C*-channel sEMG signals *x* ∈ *ℝ*^*C*^, *H* denotes a deep neural network with parameters *θ*, and *y* denotes the final gesture classification results.

The relationship between *v*_1_, *v*_2_,…, *v*_*n*_ and *x* can be formulated as

(2) $$\begin{array}{c} v_{i}=f_{vc_{i}}\left(x\right), \end{array}$$

where *f*_*vc*_*i*__,  *i*=1,2,…, *n* denotes view construction functions that generate multiview representations from raw sEMG signals.

In the field of multiview deep learning, a common approach is to build up *n* neural networks *H*_*l*_*i*__,  *i*=1,2,…, *n* to learn deep representations from *n* views, respectively, and then use a view aggregation network *H*_*a*_ to fuse the learned multiview deep representations together and obtain the final decisions *y*. Thus, equation (1) can be written as

(3) $$\begin{array}{c} y=H_{a}\left(H_{l_{1}}\left(v_{1};\theta_{l_{1}}\right),H_{l_{2}}\left(v_{2};\theta_{l_{2}}\right),\ldots ,H_{l_{n}}\left(v_{n};\theta_{l_{n}}\right);\theta_{a}\right). \end{array}$$

### 2.2. Databases

The evaluations in this work were performed offline using multichannel sEMG signals from the publicity available NinaPro databases [35]. We chose 5 subdatabases of NinaPro, which contain multichannel sEMG signals recorded from intact and transradial amputees through different types of electrodes. Details of these databases are as follows:

The first subdatabase (denoted as NinaProDB1) contains sEMG signals collected from 27 intact subjects; each subject was asked to perform 53 gestures, including 12 finger movements (denoted as Exercise A), 17 wrist movements and hand postures (denoted as Exercise B), 23 grasping and functional movement (denoted as Exercise C), and the rest movement; each gesture was repeated 10 times (i.e., 10 trials per gesture). The sEMG signals in NinaProDB1 were recorded by 10 Otto Bock 13E200-50 electrodes at a sampling rate of 100 Hz [13]. As most of the existing studies on this database excluded the rest movement for gesture recognition [10, 26, 31, 36], in our experiments we also excluded the rest movement for the convenience of performance comparison.

The second subdatabase (denoted as NinaProDB2) contains sEMG signals collected from 40 intact subjects; each subject was asked to perform 50 gestures, including Exercises B and C in NinaProDB1, 9 force patterns (denoted as Exercise D), and the rest movement; each gesture was repeated 6 times (i.e., 6 trials per gesture). The sEMG signals in NinaProDB2 were recorded by 12 Delsys Trigno Wireless electrodes at a sampling rate of 2000 Hz [13].

The third subdatabase (denoted as NinaProDB3) contains sEMG signals collected from 11 transradial amputees; each subject was asked to perform exactly the same 50 gestures as those in NinaProDB2; each gesture was repeated 6 times (i.e., 6 trials per gesture). The sEMG signals in NinaProDB3 were recorded by 12 Delsys Trigno Wireless electrodes at a sampling rate of 2000 Hz [13]. According to the authors of NinaPro database, during the sEMG recording process of NinaProDB3, three amputated subjects performed only a part of gestures due to fatigue or pain, and in two amputated subjects, the number of electrodes was reduced to ten due to insufficient space [13]. To ensure training and testing of the model can be completed, we omitted data from these subjects following the experimental configuration used by Wei et al. [31].

The fourth subdatabase (denoted as NinaProDB4) contains sEMG signals collected from 10 intact subjects; each subject was asked to perform exactly the same 53 gestures as those in NinaProDB1; each gesture was repeated 6 times (i.e., 6 trials per gesture). The sEMG signals in NinaProDB4 were recorded by the Cometa Wave Plus Wireless sEMG system with 12 electrodes, and the sampling rate was 2000 Hz [37]. After checking the data, we found that two subjects (i.e., subject 4 and subject 6) did not complete all hand movements; their data were omitted in our experiments.

The fifth subdatabase (denoted as NinaProDB5) contains sEMG signals collected from 10 intact subjects; each subject was asked to perform exactly the same 53 gestures as those in NinaProDB1; each gesture was repeated 6 times (i.e., 6 trials per gesture). Following the experimental configuration in [37], we chose 41 gestures (i.e., Exercise B and C plus rest movement) from all 53 gestures in NinaProDB5 for classification. The sEMG signals in NinaProDB5 were recorded by two Thalmic Myo armbands at a sampling rate of 200 Hz; each Myo armband contains 8 sEMG electrodes [37].

### 2.3. Data Preprocessing and View Construction

Due to memory limitation of the hardware, for experiments on NinaProDB2-DB4, we downsampled the sEMG signals from 2000 Hz to 100 Hz following the experimental configuration used in [31].

In multiview learning, view construction is usually defined as generation of multiple views from a single view of original data [38]. Considering the fairness of performance comparison, the view construction process in this paper was exactly the same as that in MV-CNN framework [31]. As a result, three different views of multichannel sEMG, denoted as *v*_1_, *v*_2_, and *v*_3_, are represented by images of discrete wavelet packet transform coefficients (DWPTC), discrete wavelet transform coefficients (DWTC), and the first Phinyomark's feature set (Phin_FS1) that are extracted from raw sEMG signals, respectively.

For the generation of the feature images, we followed the image generation algorithm proposed by Jiang and Yin [39], which is described in Algorithm 1.

Although the abovementioned three views of multichannel sEMG were proven to be the most discriminative views for gesture recognition in [31], the construction of them still requires a lot of computational time and resources, as well as their high-dimensionality results in the increase of the number of neural network parameters, making us consider the trade-off between gesture recognition accuracy and computational complexity. Thus, in this paper, we also evaluated a “two-view” configuration, which selected the two most discriminative views (i.e., *v*_1_ and *v*_2_, represented by images of DWPTC and DWTC, resp.) out of these three views of multichannel sEMG and used them as the input of the proposed HVPN framework. Details of the evaluations on the “two-view” configuration will be presented in the following sections of this paper.

For extraction of sEMG features during view construction, sliding windows were used to segment the multichannel sEMG. Early studies in MCI have pointed out that the response time of a real-time MCI system should be kept below 300 ms to avoid a time delay perceived by the user [40, 41]. For this reason, the sliding window length was set to 200 ms for most of the experiments, and the window increment was set to 10 ms except for experiments on NinaProDB5 using the sliding window length of 200 ms. For experiments on NinaProDB5 using 200 ms sliding windows, we followed the experimental configuration used by Pizzolato et al. [37] and Wei et al. [31], which set the window increment to 100 ms.

Suppose the images that represent the *i*th view have an sEMG feature dimension of *M*_*i*_ and an sEMG channel dimension of *C*, the *M*_*i*_ × *C* (width, height, respectively, depth = 1) feature space of *v*_*i*_ is firstly transformed into an *M*_*i*_ × *C* × 1 (depth, width, and height, respectively) feature space before it is input into neural network architecture of HVPN for gesture recognition. The transformation is based on the experimental results presented in [15], where the 20 × 10 × 1 (depth, width, and height, respectively) sEMG images significantly outperformed the 1 × 20 × 10 (depth, width, and height, respectively) sEMG images as the input of an end-to-end CNN in gesture recognition using 10-channel sEMG signals segmented by 20-frame sliding window.

### 2.4. The HVPN Framework

**Figure 1:**
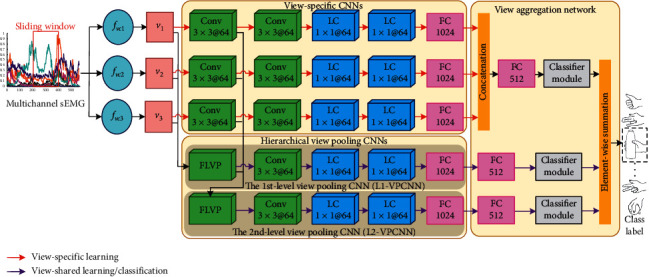
A schematic diagram of the proposed HVPN framework. FLVP, Conv, LC, and FC denote the feature-level view pooling layer, convolutional layer, locally connected layer, and fully connected layer, respectively. The numbers after the layer name denote the size and number of the filters or neurons; for example, Conv 3 × 3@64 denotes a CNN with 64 3 × 3 filters, and FC 1024 denotes an FC layer with 1024 hidden units.

A diagram of our proposed HVPN framework with all three views of multichannel sEMG is illustrated in Figure 1. The deep learning architecture of HVPN can be divided into three parts: view-specific CNNs, hierarchical view pooling CNNs, and a view aggregation network. For HVPN with the “two-view” configuration, there are two view-specific CNN branches to learn view-specific deep features from *v*_1_ and *v*_2_, respectively, and other parts are almost the same as those illustrated in Figure 1. The following sections describe the detailed network architecture and hyperparameter configurations of these parts.

### 2.5. View-Specific CNNs

After view construction, we built up three view-specific CNN branches to learn view-specific deep features from *v*_1_, *v*_2_, and *v*_3_, respectively. As shown in Figure 1, all view-specific CNN branches share the same network architecture but do not share their weights. The network architecture of each view-specific CNN branch is based on GengNet [10], which has been extensively used in sEMG-based gesture recognition [15, 31, 42]. Specifically, the images of each view are input into two convolutional layer with 64 3 × 3 filters (stride = 1), followed by two locally connected (LC) layers with 64 1 × 1 filters (stride = 1) and one fully connected (FC) layer with 1024 hidden units. For each CNN branch, we applied batch normalization and the ReLU nonlinearity function after each layer and added dropout layers to the FC layer and the last LC layer to prevent overfitting. The input of each CNN is also normalized through batch normalization.

### 2.6. Hierarchical View Pooling CNNs

**Figure 2:**
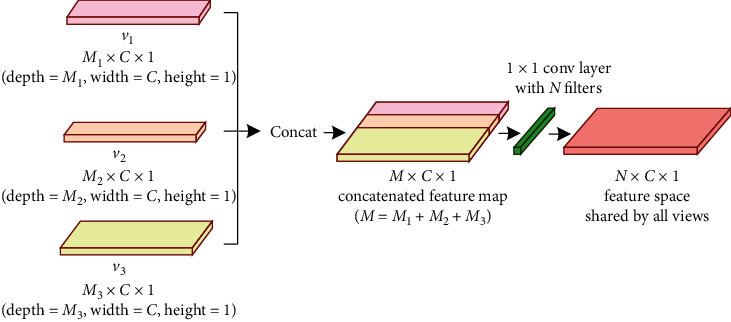
Diagram of the FLVP layer.

The hierarchical view pooling CNNs are composed of two CNN branches, namely, the first-level view pooling CNN (denoted as L1-VPCNN) and the second-level view pooling CNN (denoted as L2-VPCNN); each of them starts with an FLVP layer, which is used to learn a view-shared feature space from multiview low-level features. As illustrated in Figure 2, the FLVP layer firstly concatenates the input feature maps from different views together and then learns a unified feature space from the concatenated feature maps through a 1 × 1 convolutional layer with 64 filters. The FLVP layers in our proposed HVPN framework play two important roles: (1) each of them learns a unified feature space shared by all views from concatenated multiview low-level features for view-shared learning; (2) compared with the extensively used view pooling technique based on simple element-wise maximum [43] or average [44] operation, each FLVP layer can guarantee that its corresponding hierarchical view pooling CNN branch is deep enough to learn representative features.

Suppose we have *v*_1_ ∈ *ℝ*^*M*_1_×*C*×1^, *v*_2_ ∈ *ℝ*^*M*_2_×*C*×1^, *v*_3_ ∈ *ℝ*^*M*_3_×*C*×1^, and the multiview low-level features learned by the bottom convolutional layers of three view-specific CNN branches are $\hat{v_{1}},\hat{v_{2}},\hat{v_{3}}\in {\mathbb{R}}^{64\times C\times 1}$, respectively. The hierarchical view pooling process by FLVP layers can be formulated as follows.

The 1st-level view pooling:

(4) $$\begin{array}{c} v_{c_{1}}=v_{1}\left\|v_{2}\right\|v_{3},\\ {\hat{v}}_{l_{1}}=H_{fv_{1}}\left(v_{c_{1}};\theta_{fv_{1}}\right),\\ v_{c_{1}}\in {\mathbb{R}}^{M\times C\times 1},M=M_{1}+M_{2}+M_{3},\\ {\hat{v}}_{l_{1}}\in {\mathbb{R}}^{64\times C\times 1}. \end{array}$$

The 2nd-level view pooling:

(5) $$\begin{array}{c} v_{c_{2}}=\hat{v_{1}}\left\|\hat{v_{2}}\right\|\left.\hat{v_{3}}\right\|{\hat{v}}_{l_{1}},\\ {\hat{v}}_{l_{2}}=H_{fv_{2}}\left(v_{c_{2}};\theta_{fv_{2}}\right),\\ {\hat{v}}_{c_{2}}\in {\mathbb{R}}^{256\times C\times 1},\\ {\hat{v}}_{l_{2}}\in {\mathbb{R}}^{64\times C\times 1}, \end{array}$$

where  ‖ denotes the feature-level concatenation operation, ${\hat{v}}_{l_{i}}$ denotes the learned feature space after level-*i* view pooling, *H*_*fv*_*i*__ denotes the FLVP layer in L*i*-VPCNN, and *θ*_*fv*_*i*__ denotes its parameters.

The remaining parts of L1-VPCNN and L2-VPCNN perform view-shared learning from ${\hat{v}}_{l_{1}}$ and ${\hat{v}}_{l_{2}}$, respectively. They share the same network architecture, which is composed of one convolutional layer with 64 3 × 3 filters (stride = 1), followed by two LC layers with 64 1 × 1 filters (stride = 1) and one FC layer with 1024 hidden units.

### 2.7. View Aggregation Network

The view aggregation network is used for the following: (1) the fusion of all view-specific CNN branches and hierarchical view pooling CNN branches and (2) final gesture classification. As shown in Figure 1, the view aggregation network adopts a two-step view aggregation strategy. Specifically, it concatenates the output view-specific deep features learned by three view-specific CNN branches together at first. Then, the concatenated view-specific deep features and the view-shared deep features learned by L1-VPCNN and L2-VPCNN are input into three branches, respectively. Each branch consists of one FC layer with 512 hidden units and a classifier module, and each classifier module is composed of a G-way FC layer and a softmax classifier for gesture classification. At the top of HVPN, there is an element-wise summation operation that sums up the softmax scores predicted by all three classifier modules together to form the final classification results.

### 2.8. Evaluation Metric and Methodology

For experiments in this study, we calculated the gesture recognition accuracy for each subject as the evaluation metric, which is defined as

(6) $$\begin{array}{c} \text{accuracy}=\frac{\text{number of correct classifications}}{\text{Ttotal number of classifications}}*100\%. \end{array}$$

The evaluation methodology in this paper can be categorized into intrasubject evaluation and intersubject evaluation. Generally speaking, in intrasubject evaluation, the deep learning model is trained on a part of the data from one subject and tested on the nonoverlapping part of the data from the same subject, whereas in intersubject evaluation, the deep learning model is usually trained on data from one or a group of subjects and tested on data from another group of subjects.

For fair performance comparison, we adopted the same intrasubject and intersubject evaluation schemes as those were most commonly used in existing studies on NinaPro database [10, 13, 26, 31, 36, 42], which are described as follows.

*Intrasubject Evaluation.* For intrasubject evaluation, we followed the evaluation scheme proposed by the NinaPro team [13]. Specifically, for each subject, approximately 2/3 of the gesture trials are used as the training set; the remaining gesture trials constitute the test set. The final gesture recognition accuracy is obtained by averaging the achieved accuracy over all subjects. The selection of gesture trials for training and testing are based on the literature [13, 37].

*Intersubject Evaluation.* For intersubject evaluation, we followed the leave-one-subject-out cross-validation (LOSOCV) scheme used in the literature [31, 36, 42]. Specifically, in each fold of the cross-validation, data from one subject is used as the test set, and data from the remaining subjects is used as the training set. The final gesture recognition accuracy of the evaluation is obtained by averaging the achieved accuracy over all folds.

Specifications of the evaluation methodology on different sEMG databases are presented in Table 1.

### 2.9. Deep Domain Adaptation for Intersubject Evaluation

In intersubject evaluation, the training (i.e., source domain) and test (i.e., target domain) data comes from two nonoverlapping groups of subjects; thus, there exist distribution mismatch and domain shift across the source target domain caused by electrode shifts, changes in arm position, muscle fatigue, skin condition [45], and individual differences among subjects [46], which may dramatically degrade the classification performance of the model [47].

To reduce the negative effect of distribution mismatch and domain shift on classification performance, a number of existing deep learning based approaches [31, 42, 48] in this field have applied a novel unsupervised deep domain adaptation technique named multistream AdaBN (MS-AdaBN) [42]. The MS-AdaBN technique uses a multistream network to incrementally update the batch normalization statistics of the network training process with the calibration data.

In this work, the MS-AdaBN was also implemented for deep domain adaptation in LOSOCV, because our preliminary experiments on NinaProDB1 revealed that the LOSOCV accuracy achieved by our proposed model without deep domain adaptation is far from practical applications (i.e., <30%). Similar results were achieved by MV-CNN and reported by Wei et al. [31].

For selection of training, calibration, and test data, we followed exactly the same MS-AdaBN configuration as that used in previous works [31, 42]. It should be mentioned that as MS-AdaBN requires a relatively large amount of calibration data, it may not be the best solution for domain adaptation in the context of multichannel sEMG-based gesture recognition. Nevertheless, MS-AdaBN is not a contribution of this work, and we used it in our experiments because we wanted to ensure a fair comparison of LOSOCV accuracy between our proposed method and the previously proposed MV-CNN [31], which is a multiview deep learning framework that also adopted MS-AdaBN for domain adaptation.

## 3. Experiments

All experiments were performed offline (i.e., not real-time) on a DevMax401 workstation with NVIDIA GeForce GTX1080Ti GPU. The proposed HVPN framework was trained using the stochastic gradient descent (SGD) optimizer with 28 epochs. For all experiments, the batch size was set to 1000, and a learning rate decay strategy was adopted during training to improve convergence, which initialized the learning rate at 0.1 and divided it by 10 after 16 and 24 epochs. For all layers with dropout, the dropout rate was set to 0.65 during training.

### 3.1. Evaluation of the Hierarchical View Pooling Strategy

Evaluation of the hierarchical view pooling strategy can be divided into two steps. First, we carried an ablation study to verify the effectiveness of FLVP layer. Second, we carried out an ablation study to validate the effectiveness of the proposed hierarchical view pooling CNNs. For all experiments in these ablation studies, the sliding window length was set to 200 ms.

In the first step of the evaluation, the standard HVPN was firstly compared with its two variants, namely, HVPN-maxpool and HVPN-avgpool, on five databases (i.e., NinaProDB1-DB5). In HVPN-maxpool, the FLVP layer in L2-VPCNN was replaced by view pooling based on element-wise maximum, while in HVPN-avgpool the FLVP layer in L2-VPCNN was replaced by view pooling based on element-wise average. Meanwhile, the FLVP layers in the L1-VPCNN of HVPN-maxpool and HVPN-avgpool were retained, because the input feature spaces of L1-VPCNN have different sizes, which make it impossible for performing element-wise maximum or average operation among them.

In the second step of the evaluation, the proposed HVPN was compared with the following deep neural network architectures:

 
**VS-L1VP**: a deep network that is equivalent to HVPN without the L2-VPCNN.
 
**VS-L2VP**: a deep network that is equivalent to HVPN without the L1-VPCNN.
 
**VS-ONLY**: a deep network that only consists of view-specific CNNs, followed by a concatenation operation that fuses their output together, a FC layer with 512 hidden units and a classifier module.

**Figure 3:**
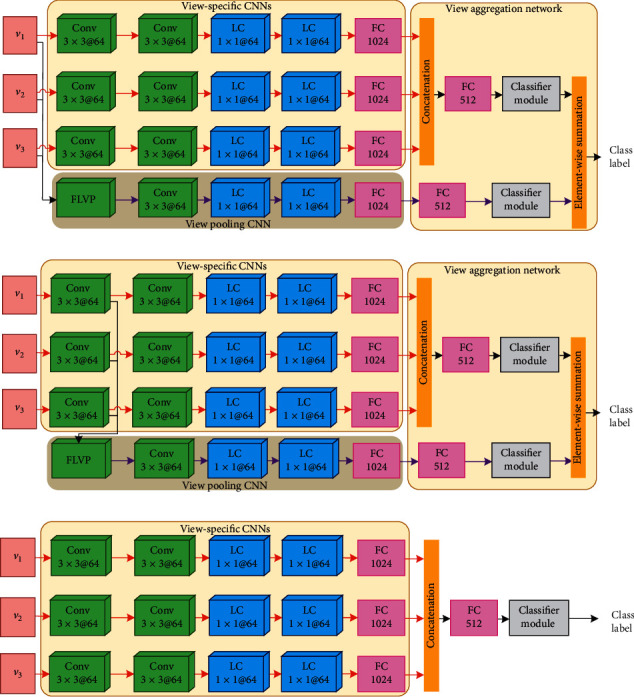
Schematic diagrams of (a) VS-L1VP, (b) VS-L2VP, and (c) VS-ONLY.

The schematic illustration of VS-L1VP, VS-L2VP, and VS-ONLY is depicted in Figure 3. Compared to HVPN that contains hierarchical view pooling CNNs, there is only one view pooling CNN in VS-L1VP, as well as VS-L2VP, for view-shared learning.

### 3.2. Comparison with Related Works

The gesture recognition accuracy achieved by the proposed HVPN framework, as well as the gesture recognition accuracy achieved by the proposed HVPN framework with the “two-view” configuration (denoted as HVPN-2-view), was further compared with related works on five databases (i.e., NinaProDB1-DB5). For the aim of fairness in this comparison, among various machine learning methods that were proposed for sEMG-based gesture recognition and tested on NinaPro, we only considered the ones that meet the following requirements: (1) their reported gesture recognition accuracy was achieved using exactly the same intrasubject or intersubject gesture recognition schemes as described in Section 2; (2) the input of their machine learning models were engineered features, not raw sEMG signals.

To prevent overfitting, a pretraining strategy that has been widely used by the compared methods [26, 31] was also adopted in this work. Specifically, for each experiment, a pretrained model was firstly trained using all available training data; then, the gesture recognition model for each subject was initialized by the pretrained model. For all layers with dropout, the dropout rate was set to 0.5 during the pretraining stage.

For comparison of intrasubject gesture recognition accuracy, we evaluated the gesture recognition accuracy achieved with 50 ms, 100 ms, 150 ms, and 200 ms sliding windows. Moreover, the gesture recognition accuracy obtained by majority voting on all 200 ms windows within each trial is also presented in the column labeled “Trial.” For comparison of LOSOCV (i.e., intersubject gesture recognition) accuracy, we only evaluated the gesture recognition accuracy achieved with 200 ms sliding windows.

## 4. Results and Discussion

### 4.1. Multichannel sEMG-Based Gesture Recognition Enhanced by Hierarchical View Pooling

Table 2 presents the intrasubject and LOSOCV accuracy achieved by the standard HVPN, HVPN-maxpool, and HVPN-avgpool on five databases. The proposed HVPN framework achieved the intrasubject gesture recognition accuracy of 86.8%, 84.4%, 68.2%, 70.8%, and 88.6% on NinaProDB1, DB2, DB3, DB4, and DB5, respectively, and achieved the LOSOCV accuracy of 83.1%, 79.0%, 65.6%, 67.0%, and 87.1% on NinaProDB1, DB2, DB3, DB4, and DB5, respectively. The gesture recognition accuracy achieved by HVPN was higher than that achieved by HVPN-maxpool and HVPN-avgpool in all experiments, indicating that the FLVP layer can achieve better gesture recognition accuracy than the conventional view pooling approaches based on element-wise maximum or average operation. However, when evaluated on NinaProDB1, DB2, DB3, and DB4, the performance improvement brought by the FLVP layer was subtle (i.e., from +0.2% to +0.4% over element-wise max or average pooling). This is likely due to the fact that in HVPN-maxpool and HVPN-avgpool we only replaced the FLVP layer in L2-VPCNN with conventional view pooling, making them very similar to the original HVPN.

Table 3 presents the intrasubject and LOSOCV accuracy achieved by HVPN, VS-L1VP, VS-L2VP, and VS-ONLY on five databases (i.e., NinaProDB1-DB5). According to the experimental results in Table 3, the deep neural network architectures with view pooling CNNs (i.e., HVPN, VS-L1VP, and VS-L2VP) significantly outperformed VS-ONLY, indicating that combining view-specific learning with view-shared learning is better than performing view-specific learning alone in the context of multiview deep learning for multichannel sEMG-based gesture recognition. Moreover, the intrasubject and LOSOCV accuracy achieved by HVPN was higher than that achieved by VS-L1VP and VS-L2VP on all databases, which proves the effectiveness of our proposed hierarchical view pooling strategy in improving gesture recognition accuracy.

### 4.2. Comparison with Related Works Based on Intrasubject Evaluation

Table 4 presents the intrasubject gesture recognition accuracy achieved by various methods on the first five subdatabases of NinaPro. Among these methods, the methods proposed in [13, 36, 37] are shallow learning frameworks, the methods proposed in [25–27, 49, 50] are single-view deep learning frameworks, and the method proposed in [31] is a multiview deep learning framework (i.e., MV-CNN). All the above-mentioned methods are non-end-to-end methods using engineered sEMG features as their input, and they used exactly the same intrasubject evaluation scheme as that was used in our work.

Experimental results in Table 4 demonstrate that when using all three views of multichannel sEMG as input, the proposed HVPN achieved the intrasubject gesture recognition accuracy of 88.4%, 85.8%, 68.2%, 72.9%, and 90.3% on NinaProDB1, DB2, DB3, DB4, and DB5, respectively, with the sliding window length of 200 ms, which outperformed not only shallow learning frameworks [13, 36, 37] but also deep learning frameworks [25, 26, 31, 49, 50] that were proposed for sEMG-based gesture recognition in recent years.

Compared to MV-CNN, which is also a multiview deep learning framework, experimental results show the following: (1) when using exactly the same input, the gesture accuracy achieved by MV-CNN was significantly inferior to that achieved by HVPN on all databases; (2) when the number of input views of HVPN was reduced to two (i.e., denoted as HVPN-2-view in Table 4), it still outperformed MV-CNN framework on most of the databases (i.e., NinaPro DB2, DB3, DB4, and DB5), and their gesture recognition accuracy on NinaProDB1 was almost the same. For example, when the sliding window length was set to 200 ms, the HVPN-2-view achieved the intrasubject gesture recognition accuracy of 88.1%, 85.0%, 67.9%, 72.1%, and 90.1% on NinaPro DB1, DB2, DB3, DB4, and DB5, respectively. By comparison, the intrasubject gesture recognition accuracy achieved by MV-CNN on NinaPro DB1, DB2, DB3, DB4, and DB5 was 88.2%, 83.7%, 64.3%, 54.3%, and 90.0%, respectively. These results indicate that compared to MV-CNN, the HVPN framework can achieve better or similar intrasubject gesture recognition accuracy using less input data.

We also found that the intrasubject gesture recognition accuracy achieved by MV-CNN on NinaPro DB4 was much lower than that achieved by a shallow learning method (i.e., random forests [37]). By comparison, our proposed HVPN achieved the intrasubject gesture recognition accuracy of 72.9% on NinaPro DB4, with the sliding window length of 200 ms, which significantly outperformed both MV-CNN [31] and the random forests-based method [37].

### 4.3. Comparison with MV-CNN Based on Intersubject Evaluation

As very few studies in this field have presented the LOSOCV accuracy of recognizing all gestures in any of the NinaPro subdatabases, considering the difference in evaluation methodology and domain adaptation strategy, in this section, we focused on comparison with the MV-CNN framework [31], which used exactly the same intersubject evaluation scheme and domain adaptation technique as our proposed HVPN framework.

The LOSOCV accuracy achieved by MV-CNN and our proposed HVPN framework on five databases is presented in Table 5. The MV-CNN framework achieved the LOSOCV accuracy of 84.3%, 80.1%, 55.5%, 52.6%, and 87.2% on NinaProDB1, DB2, DB3, DB4, and DB5, respectively, with the sliding window length of 200 ms. By comparison, the HVPN framework achieved the LOSOCV accuracy of 84.9%, 82.0%, 65.6%, 70.2%, and 88.9% on NinaPro DB1, DB2, DB3, DB4, and DB5, respectively, with the sliding window length of 200 ms, which significantly outperformed MV-CNN. Similar to the results of intrasubject evaluation, the LOSOCV accuracy achieved by HVPN framework with the “two-view” configuration (i.e., denoted as HVPN-2-view in Table 5) also outperformed that achieved by MV-CNN framework on all databases, indicating that HVPN framework can achieve better LOSOCV accuracy than MV-CNN using less input data.

## 5. Conclusions

This paper proposed and implemented a hierarchical view pooling network (HVPN) framework, which improves multichannel sEMG-based gesture recognition by not only view-specific learning under each individual view but also view-shared learning in feature spaces that are hierarchically pooled from multiview low-level features.

Ablation studies were conducted on five multichannel sEMG databases (i.e., NinaPro DB1–DB5) to validate the effectiveness of the proposed framework. Results show the following: (1) when the FLVP layer in L2-VPCNN was replaced by conventional view pooling based on element-wise max pooling or average pooling, both intrasubject and LOSOCV accuracy degraded; (2) the proposed HVPN outperformed its two simplified variants that have only one view pooling CNN, as well as a deep neural network architecture that only consists of view-specific CNNs, in both intrasubject evaluation and LOSOCV. According to the above results, the effectiveness of the proposed hierarchical view pooling strategy can be proven.

Furthermore, we carried out performance comparison with the state-of-the-art methods on five databases (i.e., NinaPro DB1–DB5). Experimental results have demonstrated the superiority of the proposed HVPN framework over other deep learning and shallow learning-based methods. When using sliding windows of 200 ms, the proposed HVPN achieved the intrasubject gesture recognition accuracy of 88.4%, 85.8%, 68.2%, 72.9%, and 90.3% on NinaPro DB1, DB2, DB3, DB4, and DB5, respectively. The LOSOCV accuracy achieved on NinaPro DB1, DB2, DB3, DB4, and DB5 using 200 ms sliding windows was 84.9%, 82.0%, 65.6%, 70.2%, and 88.9%, respectively.

Limited by experimental conditions, we only considered offline experiments to verify our proposed HVPN framework. Our future work will focus on online evaluation of the proposed multiview deep learning framework. Moreover, in the future, we will investigate the integration of our proposed framework with hardware systems, such as upper-limb prostheses [51, 52] and space robots [53, 54] that are driven by multichannel sEMG signals.

## Figures and Tables

**Algorithm 1:**
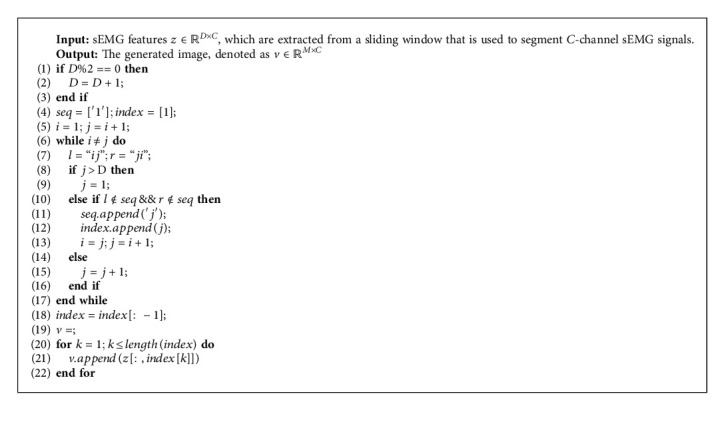
The image generation algorithm used in this paper [39].

**Table 1:** Specifications of the evaluation methodology on different sEMG databases.

Databases	Intrasubject		Intersubject
	Trials for training	Trials for testing	

NinaPro DB1	1st, 3rd, 4th, 6th, 7th, 8th, 9th	2nd, 5th, 10th	LOSOCV
NinaPro DB2	1st, 3rd, 4th, 6th	2nd, 5th	LOSOCV
NinaPro DB3	1st, 3rd, 4th, 6th	2nd, 5th	LOSOCV
NinaPro DB4	1st, 3rd, 4th, 6th	2nd, 5th	LOSOCV
NinaPro DB5	1st, 3rd, 4th, 6th	2nd, 5th	LOSOCV

**Table 2:** Gesture recognition accuracy achieved by the standard HVPN, HVPN-maxpool, and HVPN-avgpool on five databases.

Database	Evaluation methodology	HVPN	HVPN-maxpool	HVPN-avgpool

NinaProDB1	Intrasubject	**86.8%**	86.4%	86.5%
NinaProDB2	Intrasubject	**84.4%**	84.1%	84.1%
NinaProDB3	Intrasubject	**68.2%**	68.0%	67.9%
NinaProDB4	Intrasubject	**70.8%**	70.5%	70.5%
NinaProDB5	Intrasubject	**88.6%**	88.1%	88.1%

NinaProDB1	LOSOCV	**83.1%**	82.7%	82.8%
NinaProDB2	LOSOCV	**79.0%**	78.8%	78.7%
NinaProDB3	LOSOCV	**65.6%**	65.4%	65.3%
NinaProDB4	LOSOCV	**67.0%**	66.6%	66.6%
NinaProDB5	LOSOCV	**87.1%**	86.4%	86.6%

Results in bold entries indicate best performance.

**Table 3:** Gesture recognition accuracy achieved by HVPN, VS-L1VP, VS-L2VP, and VS-ONLY on five databases.

Database	Evaluation methodology	HVPN	VS-L1VP	VS-L2VP	VS-ONLY

NinaProDB1	Intrasubject	**86.8%**	86.5%	86.2%	85.8%
NinaProDB2	Intrasubject	**84.4%**	84.1%	83.9%	83.4%
NinaProDB3	Intrasubject	**68.2%**	67.7%	67.5%	67.2%
NinaProDB4	Intrasubject	**70.8%**	69.9%	69.7%	68.5%
NinaProDB5	Intrasubject	**88.6%**	87.9%	88.3%	87.2%

NinaProDB1	LOSOCV	**83.1%**	82.6%	82.5%	81.9%
NinaProDB2	LOSOCV	**79.0%**	78.7%	78.7%	78.1%
NinaProDB3	LOSOCV	**65.6%**	65.5%	65.0%	64.7%
NinaProDB4	LOSOCV	**67.0%**	66.3%	65.7%	65.2%
NinaProDB5	LOSOCV	**87.1%**	86.2%	86.5%	84.7%

Results in bold entries indicate best performance.

**Table 4:** Intrasubject gesture recognition accuracy in comparison with related works on five databases.

Machine learning (ML) model	Type of ML model	Input of ML model	Database	Num. of gestures for classification	Window length				
					50 ms	100 ms	150 ms	200 ms	Trial

Random forests [13]	Shallow learning	5 hand-crafted features	NinaProDB1	50	N.A.	N.A.	N.A.	75.3%	N.A.
Dictionary learning [36]	Shallow learning	MLSVD-based features	NinaProDB1	52	N.A.	N.A.	N.A.	N.A.	97.4%
HuNet [26]	CNN-RNN	Phinyomark feature set	NinaProDB1	52	N.A.	N.A.	86.8%	87.0%	97.3%
MV-CNN [31]	Multiview CNN	3 views of sEMG	NinaProDB1	52	85.8%	86.8%	87.4%	88.2%	N.A.
ChengNet [49]	CNN	Multi-sEMG-features image	NinaProDB1	52	N.A.	N.A.	N.A.	82.5%	N.A.
**HVPN-2-view**	**Multi-view CNN**	**2 views of sEMG**	**NinaProDB1**	**52**	**85.4%**	**86.5%**	**87.2%**	**88.1%**	**97.8%**
**HVPN**	**Multi-view CNN**	**Same as** [31]	**NinaProDB1**	**52**	**86.0%**	**86.9%**	**87.7%**	**88.4%**	**98.0%**

Random forests [13]	Shallow learning	Hand-crafted features	NinaProDB2	50	N.A.	N.A.	N.A.	75.3%	N.A.
ZhaiNet [25]	CNN	sEMG spectrogram	NinaProDB2	50	N.A.	N.A.	N.A.	78.7%	N.A.
HuNet [26]	CNN-RNN	Phinyomark feature set	NinaProDB2	50	N.A.	N.A.	N.A.	82.2%	97.6%
MV-CNN [31]	Multiview CNN	3 views of sEMG	NinaProDB2	50	80.6%	81.1%	82.7%	83.7%	N.A.
**HVPN-2-view**	**Multiview CNN**	**2 views of sEMG**	**NinaProDB2**	**50**	**82.7%**	**83.8%**	**83.3%**	**85.0%**	**97.8%**
**HVPN**	**Multiview CNN**	**Same as** [31]	**NinaProDB2**	**50**	**82.3%**	**84.1%**	**84.8%**	**85.8%**	**98.1%**

Support vector machine (SVM) [13]	Shallow learning	5 hand-crafted features	NinaProDB3	50	N.A.	N.A.	N.A.	46.3%	N.A.
MV-CNN [31]	Multiview CNN	3 views of sEMG	NinaProDB3	50	N.A.	N.A.	N.A.	64.3%	N.A.
ED-TCN [27]	TCN	MAV sequences	NinaProDB3	41	N.A.	N.A.	63.5%	N.A.	N.A.
**HVPN-2-view**	**Multiview CNN**	**2 views of sEMG**	**NinaProDB3**	**50**	**64.4%**	**65.7%**	**66.8%**	**67.9%**	**80.3%**
**HVPN**	**Multiview CNN**	**Same as** [31]	**NinaProDB3**	**50**	**64.5%**	**65.9%**	**66.9%**	**68.2%**	**80.7%**

Random forests [37]	Shallow learning	mDWT features	NinaProDB4	53	N.A.	N.A.	N.A.	69.1%	N.A.
MV-CNN [31]	Multiview CNN	3 views of sEMG	NinaProDB4	53	N.A.	N.A.	N.A.	54.3%	N.A.
**HVPN-2-view**	**Multiview CNN**	**2 views of sEMG**	**NinaProDB4**	**53**	**60.1%**	**63.2%**	**67.6%**	**72.1%**	**81.1%**
**HVPN**	**Multiview CNN**	**Same as** [31]	**NinaProDB4**	**53**	**58.3%**	**67.1%**	**70.5%**	**72.9%**	**81.7%**

SVM [37]	Shallow learning	mDWT features	NinaProDB5	41	N.A.	N.A.	N.A.	69.0%	N.A.
ShenNet [50]	Stacking-based CNN	TD, FD and TFD features	NinaProDB5	40	N.A.	N.A.	N.A.	72.1%	N.A.
MV-CNN [31]	Multiview CNN	3 views of sEMG	NinaProDB5	41	N.A.	N.A.	N.A.	90.0%	N.A.
**HVPN-2-view**	**Multiview CNN**	**2 views of sEMG**	**NinaProDB5**	**41**	**88.7%**	**89.1%**	**89.9%**	**90.1%**	**98.8%**
**HVPN**	**Multiview CNN**	**Same as** [31]	NinaProDB5	**41**	**88.7%**	**89.3%**	**90.0%**	**90.3%**	**98.4%**

N.A. denotes not applicable, and bold entries indicate our proposed method. HVPN-2-view refers to the proposed HVPN framework with the “two-view” configuration (i.e., using *v*_1_ and *v*_2_ as its input). †It should be mentioned that existing MCIs seldom segment raw sEMG signals by trial due to the constraint that the maximal response time of an MCI should be kept below 300 ms [40, 41]. ‡For experiments on HVPN, the predicted class label of each gesture trial is obtained by majority voting on all 200 ms sliding windows within it.

**Table 5:** LOSOCV accuracy in comparison with MV-CNN on five databases.

ML model	Type of ML model	Domain adaptation method	Database	Num. of gestures for classification	LOSOCV accuracy (achieved with 200 ms window)

MV-CNN [31]	Multiview CNN	MS-AdaBN	NinaProDB1	52	84.3%
**HVPN-2-view**	**Multiview CNN**	**MS-AdaBN**	**NinaProDB1**	**52**	**84.5%**
**HVPN**	**Multiview CNN**	**MS-AdaBN**	**NinaProDB1**	**52**	**84.9%**

MV-CNN [31]	Multiview CNN	MS-AdaBN	NinaProDB2	50	80.1%
**HVPN-2-view**	**Multiview CNN**	**MS-AdaBN**	**NinaProDB2**	**50**	**81.8%**
**HVPN**	**Multiview CNN**	**MS-AdaBN**	**NinaProDB2**	**50**	**82.0%**

MV-CNN [31]	Multiview CNN	MS-AdaBN	NinaProDB3	50	55.5%
**HVPN-2-view**	**Multiview CNN**	**MS-AdaBN**	**NinaProDB3**	**50**	**65.4%**
**HVPN**	**Multiview CNN**	**MS-AdaBN**	**NinaProDB3**	**50**	**65.6%**

MV-CNN [31]	Multiview CNN	MS-AdaBN	NinaProDB4	53	52.6%
**HVPN-2-view**	**Multiview CNN**	**MS-AdaBN**	**NinaProDB4**	**53**	**69.9%**
**HVPN**	**Multiview CNN**	**MS-AdaBN**	**NinaProDB4**	**53**	**70.2%**

MV-CNN [31]	Multiview CNN	MS-AdaBN	NinaProDB5	41	87.2%
**HVPN-2-view**	**Multiview CNN**	**MS-AdaBN**	**NinaProDB5**	**41**	**88.8%**
**HVPN**	**Multiview CNN**	**MS-AdaBN**	**NinaProDB5**	**41**	**88.9%**

N.A. denotes not applicable, and bold entries indicate our proposed method. HVPN-2-view refers to the proposed HVPN framework with the “two-view” configuration (i.e., using *v*_1_ and *v*_2_ as its input).

## Data Availability

The multichannel sEMG data supporting the findings of this study are from the NinaPro dataset, which is publicly available at http://ninapro.hevs.ch. Papers describing the NinaPro dataset are cited at relevant places within the text as references [13, 37]. The processed data and trained deep learning models used to support the findings of this study are available from the corresponding author upon request.
